# The impact of status and social context on health service co-design: an example from a collaborative improvement initiative in UK primary care

**DOI:** 10.1186/s12874-018-0608-5

**Published:** 2018-11-16

**Authors:** Ian Litchfield, Louise Bentham, Ann Hill, Richard J. McManus, Richard Lilford, Sheila Greenfield

**Affiliations:** 1 Institute of Applied Health Research, University of Birmingham, Birmingham, UK; 2 Worcestershire Acute Hospitals NHS Trust, Worcester, UK; 3 Nuffield Department of Primary Care Health Sciences, University of Oxford, Oxford, UK; 4 Warwick Centre for Applied Health Research and Delivery, Warwick Medical School, University of Warwick, Coventry, UK

**Keywords:** Focus groups, Healthcare, teamwork, Healthcare, primary, Healthcare users’ experience, Relationships, patient-provider

## Abstract

**Background:**

Increasingly, collaborative participatory methods requiring open and honest interaction between a range of stakeholders are being used to improve health service delivery. To be successful these methodologies must incorporate perspectives from a range of patients and staff. Yet, if unaccounted for, the complex relationships amongst staff groups and between patients and providers can affect the veracity and applicability of co-designed solutions.

**Methods:**

Two focus groups convened to discuss suggestions for the improvement of blood testing and result communication in primary care. The groups were mixed of patients and staff in various combinations drawn from the four participating study practices. Here we present a secondary mixed-method analysis of the interaction between participants in both groups using sociogrammatic and thematic analysis.

**Results:**

Despite a similar mix of practice staff and patients the two groups produced contrasting discussions, seemingly influenced by status and social context. The sociograms provided a useful insight into the flow of conversation and highlighted the dominance of the senior staff member in the first focus group. Within the three key themes of social context, the alliances formed between participants and the fluidity of the roles assumed manifested differently between groups apparently dictated by the different profile of the participants of each.

**Conclusions:**

For primary care service improvement attention must be paid to the background of participants when convening collaborative service improvement groups as status and imported hierarchies can have significant connotations for the data produced.

## Background

Over the last decade, healthcare has seen a move toward greater teamwork across traditional staff and patient boundaries [1–3] and more recently reflected in a similar shift in quality improvement strategies. Where previously the tendency was to privilege clinical expertise over the subjective knowledge of patients and non-clinical staff [4–6], there is now recognition of the positive impact of mobilising a broader range of expertise in improving service delivery [7]. One way this can be achieved is by utilising the experience of patients, alongside those of a range of clinical and non-clinical staff to co-design service improvement interventions [8]. This type of collaborative approach is now increasingly being utilised in health care organisations across the globe in both primary and secondary care settings [9, 10].

A central principle of co-design is the provision of a platform where all participants are able to express themselves openly, question existing systems and methods of working, and explore alternative perspectives [11]. In this way, interventions are developed born of the experience of all stakeholders and acknowledging their preferences and needs [1, 12, 13]. However, establishing uninhibited working groups of patients and staff is not necessarily straightforward [14], participants often have a range of motivations for becoming actively involved [15] and the complex relationship between academics, NHS staff, patients and the public is rarely considered [16]. Previous studies have explored how characteristics of participants, can influence the complex social context of heterogeneous focus groups analogous to those used in co-design [17, 18]. Pressures of social desirability can induce participants to offer only certain information or play particular roles, [19–21] and alliances are formed, influenced by the degree of familiarity amongst participants. Constituting collaborative groups requires careful consideration, previous work has described the advantages to pre-existing groups [17], for example, they’re easier to recruit and their familiarity facilitates discussion. Stranger groups whilst harder to recruit can promote greater disclosure as challenges to others can be more probing without fear of repercussion [22, 23]. In service improvement participants may or may not be known to each other depending on the scope, setting, and scale of the project. In primary care settings where continuity of care is greater and organisations are smaller participants, whether staff or patients, are more likely to be familiar. Existing advice on recruiting extends only to recruiting a range of appropriate participants [24, 25], there has been no explicit discussion as to how the prior relationship of participants might influence the outcomes of co-design initiatives.

The TRaCKED study was a multiphase project that used co-design principles to source, implement and evaluate improvements in the blood test and result communication process in UK primary care [26]. As part of this process we convened two groups mixed of patients and staff used to refine emergent ideas for improvement. Here we present the results of secondary analysis and the particular combination of staff and patients influenced the discussions of each leading to contrasting experiences for participants and ultimately influencing service improvement outputs.

## Methods

### Testing result communication: Knowledge evaluation and development study (TRaCKED)

Successful management of test results within primary care in the UK is hindered by the fragmented setting and an absence of satisfactory guidelines [27]. In response, we worked with patients and staff to develop and implement appropriate interventions to improve the testing and result communication process [28, 29]. In doing so, two focus groups mixed of patients and staff were convened to refine suggestions for improving the process.

### Settings and participants

The study was set in four purposively selected general practices of different size, and socio-economic environment located within the West Midlands (UK). The staff and patient participants had recent experience of the testing and result communication process and in attempt to create groups of maximum variability we invited patients with a range of age, gender and ethnicity and staff with a variety of seniority and clinical and non-clinical roles to participate.

### Focus groups

Each focus group was facilitated by the same experienced moderator with a professional background in health service redesign (AH). Identical topic guides and visual aids were used in both discussions which were digitally recorded and transcribed verbatim.

### Analysis

Here we present a secondary analysis of the data which consisted of both a quantitative and qualitative element. For the quantitative analysis, we used word counts to create sociograms to produce a visual representation of the group dynamic [30]. The widths of the arrows were directly proportionate to the number of times the conversation passed from one individual to another. In this way they demonstrate the flow of conversation, helping us identify patterns of communication, recognise alliances, and facilitating comparison between groups [31]. The qualitative element consisted of a thematic analysis where each transcript was read and the findings analysed thematically by three of the authors (IL, LB, SG) who met and agreed emerging themes to decide on a coding framework for data on group interaction. This interaction data was analysed using the same approach as individual or group data; emerging themes were identified and examples from the transcripts selected [32].

### Ethics, consent and permissions

This study was given favourable opinion by the National Research Committee of West Midlands - The Black Country and by the Birmingham and Black Country Comprehensive Local Research Network (REC reference number: 10/H1202/71). All patient and staff participants provided written informed consent to participate in the study.

## Results

The characteristics of the four practices from which the focus group participants were drawn are shown in Table 1. The largest had a patient list size in access of some 27,000 and the smallest just over 7000 with a range of IMD scores [33]. A total of 8 staff and 7 patients attended the groups. No GPs were able to attend and the most senior staff present at each were non-clinical (Table 2). Within Focus Group One (FG1) there were six members of staff and four patients. In Focus Group 2 (FG2) there were two staff and three patients. The characteristics and affiliations of participants are described in Table 1.

**Table 1:** Characteristics of participating practices

Practice Characteristics	Practice 1	Practice 2	Practice 3	Practice 4
Number of GPs (fte)^a^	7.3	3.0	6.3	12.3
IMD Ranking^b^	15,066	13,866	871	7127
Number of patients	23,727	8447	7059	27,430

^a^Full time equivalent

^b^The IMD codes [33] produced by the UK government and first released in 2004, provide indicators of deprivation in local authority areas to inform health and social policy. The higher the ranking the more deprived the area

**Table 2:** **N**umber of words contributed by each focus group participant

	Practice	Number of words (% of total)
Focus Group 1 (FG1)		
Practice Manager (PM-1)	Practice 1	4121 (40%)
Patient (Pt1–1)	Practice 1	1239 (12.0%)
IT Lead (IT-1)	Practice 1	1156 (11.2%)
Patient (Pt-4)	Practice 4	1020 (10.0%)
Lead Receptionist (LR-1)	Practice 1	908 (8.8%)
Lead Receptionist (LR-2)	Practice 2	399 (3.9%)
Practice secretary (PS-2)	Practice 2	361 (3.5%)
Phlebotomist (Pbt-3)	Practice 3	537 (5.0%)
Patient (Pt-2)	Practice 2	405 (4.0%)
Patient (Pt −3)	Practice 3	161 (1.6%)
	Total Word Count FG1	10,370 (100%)
Focus Group 2 (FG2)		
Office Manager (OM-3)	Practice 3	2959 (33.3%)
Research Nurse (Res Nrs-3)	Practice 3	2261 (25.4%)
Patient (Pt-4)	Practice 4	1666 (18.8%)
Patient (Pt2–1)	Practice 1	1156 (13.0%)
Patient (Pt3–1)	Practice 1	840 (9.5%)
	Total Word Count FG2	8882 (100%)

### Word counts and sociogrammatical analysis

**Fig. 1:**
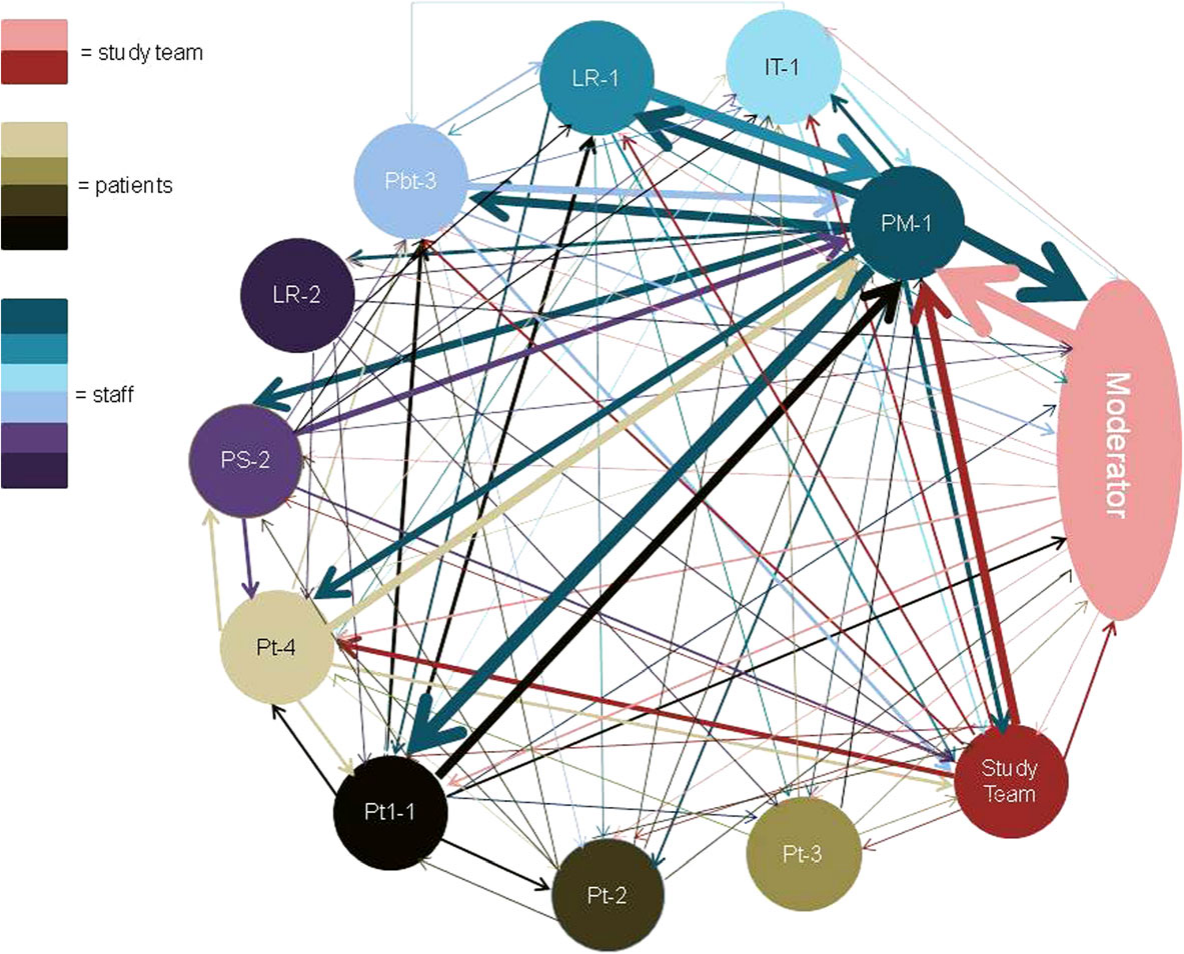
Sociogram Design Group 1

For each participant a word count was produced indicating the contribution of each member of the group (see Table 2) which allowed the production of sociograms. In Focus Group 1 (FG1) with no seating plan in place, staff sat together on one side of the table and patients, the other (see Fig. 1). The majority of the moderator’s questions and prompts were picked up by the practice manager. It’s also notable how the comments of other staff were most frequently addressed to or answered by the practice manager.

**Fig. 2:**
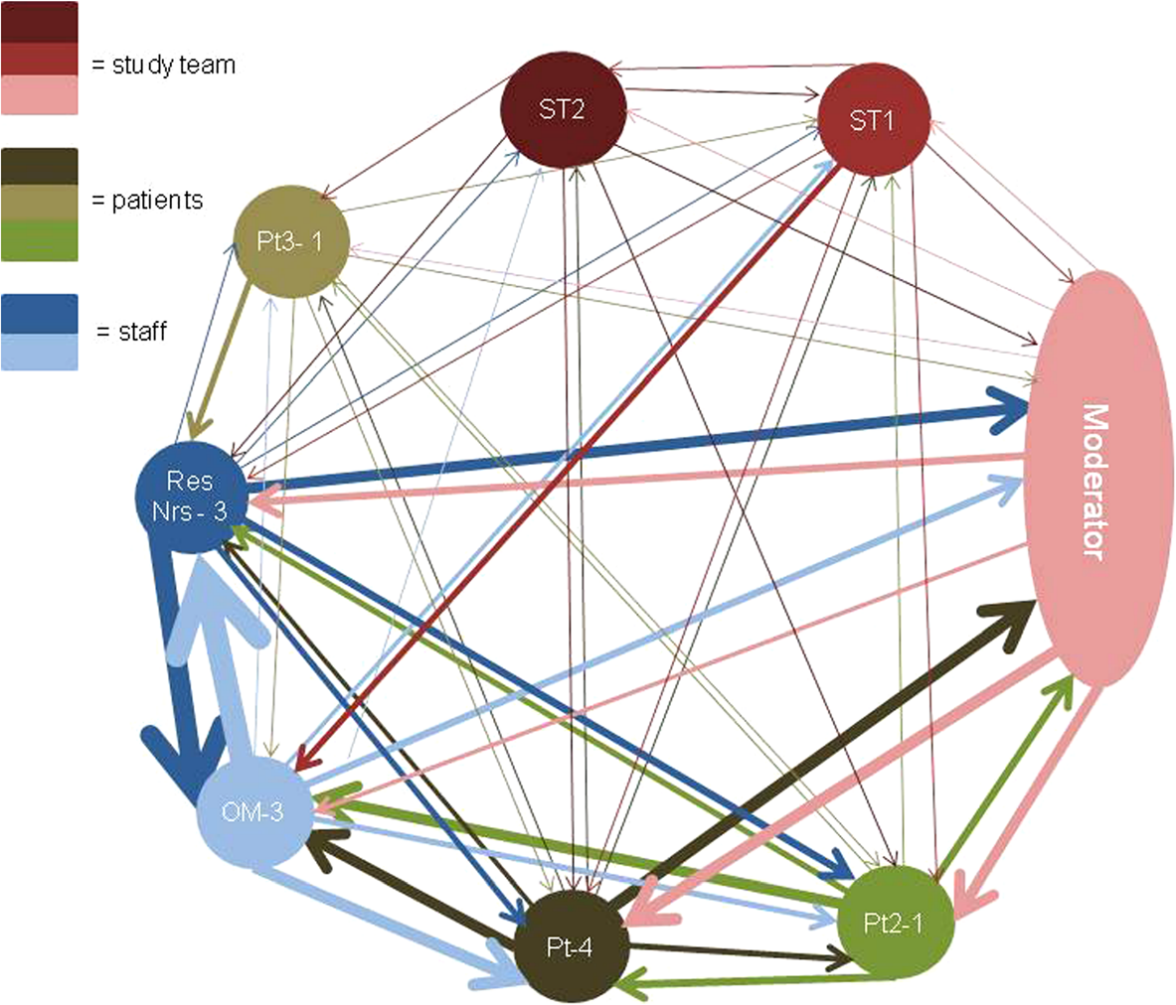
Sociogram Design Group 2

In Focus Group 2 (FG2) the sociogram showed evidence of an alliance between staff participants from the same practice, though the conversation appears to be shared more equally around the group (see Fig. 2). The word counts showed that a patient representative was more voluble and spoke a similar number of words to staff and moderator in contrast to the quieter patients in FG1.

### Thematic analysis

Three key themes emerged that described the interactions between participants, though there were key differences in how they manifested across groups as summarised in Table 3. The three themes are, first social context namely status and associational contexts describing the influence of the status of individual members and the underlying association between group members respectively; second, the alliances that formed amongst focus group members either within staff groups or between staff and patient participants; and third the assumption of role within each group and whether the roles assumed by participants was fixed or fluid. These are described in further detail below and exemplified by passages taken from the transcripts.

**Table 3:** Key themes by focus group

Theme	*Focus Group 1*	Focus Group 2
Social context	*Status*	*Associational*
Alliances amongst focus group members	*Between staff*	*Between staff and patients*
Assumption of role within the group	*Fixed role assumption*	*Fluid role assumption*

## Social context

The social context describes the dominating influence on the group discussion and can depend upon a number of influences. In our two focus groups, the key influences on contexts were status, referring to the relative positions of the participants in local hierarchies [17] and associational, which relates to the common characteristic that brings the group together [17].

### Status

The status of individual practice staff and the hierarchical influence of their workplace appeared to influence the course of the discussion within FG1. This was evidenced by the way the practice manager, the most senior member of staff present, would seem to occupy the dominant position in the group. They were frequently the first to respond to or be addressed by both the moderator and fellow participants. Sometimes they would interrupt other members of the group on the occasion when they were first to respond to the moderator. In the example below she spoke over the receptionist from her practice to confirm the patient’s role in communicating results.

Moderator (Mod): Until your sample is taken, or the patient’s sample is taken, is there an appropriate amount of time?

Lead receptionist, practice one (LR-1): We normally ask patients to ri…

Practice manager, practice one (PM-1): ask them to ring back after a week

In another example of this dominance the practice manager appeared to influence the input of staff from other practices. Here the phlebotomist from Practice 3 appears to change her mind over the course of the conversation to fall in line with the practice manager’s viewpoint on same day phlebotomy appointments.

Mod: So how and what would you like to do differently?

Phlebotomist, practice three (Pbt-3): I’d like if they could have blood tests on the same day, save the patients coming back

PM-1: …we’re not a bottomless pit, this is the NHS and in the ideal world wouldn’t it be great just ‘Oh yeah, take a seat over there’

Pbt-3: Yeah, but with…

PM-1: ‘We’ll take your blood’ you know…you’ve got to think that there’s immunisations going on, there’s dressings, daily people are having dressings, stitches out. The nurses aren’t, and health care assistants, aren’t just taking blood are they?

Pbt-3: No, no.

### Associational context

In FG2, the individuals appeared more aware of their common association i.e. to discuss ways in which existing systems might be improved. For example, when the idea of informing all patients of normal results was discussed, it received a receptive approach from staff in collaboration with patients.

Office manager 3: I agree with informing patients.

Patient, practice 4: Whatever system you have, there’s got to be a mistake proof method that encompasses all the means of communication; text, telephone, written… it’s all got to be mistake proof.

Office manager 3: I think as you say, they should actually, almost, they should have a copy of their results. That would be the ideal way.

Mod: Aim for zero errors.

Research nurse, practice 3: But if you…used this cytology model for example if the consortiums are going out and saying to people ‘we’re buying in more services, we’re going to use your results service, and in two weeks we expect every patient who had this test done to be given ‘Your blood test is normal’ or ‘Your blood tests are abnormal, if abnormal, this is what we do’ you know, If you can do it, [for cytology] - I know cytology’s got a smaller group of people - but it’s already set up isn’t it?

As the conversation continued staff and patients remained mutually supportive though not at the expense of practical considerations. In this example the research nurse served a reminder that any amendment would have to be quick and easy to use due to the pressures GPs were under.

Res Nrs-3: You can almost, actually when doctors go in and pick up the test results transferred from the labs, it would be very easy at that juncture to put in a standard letter, press the button for ‘standard letter’ and get it printed off, so…

Patient, practice 2 (Pt-2): Or send a text, or whatever it is.

Pt-4: Whatever the patient prefers.

OM-3: Yeah, maybe look at it that way, yeah.

Res Nrs-3: As long it’s … as long as the doctor doesn’t spend time looking for it, it has to be quite a fast system, because they are pushed for time they really … do have a lot to do of course … and we have to make it as easy as possible for it to be used correctly.

## Alliances amongst focus group members

Another aspect of focus group interaction is the formation of alliances between group members (REF), the nature of these varied between the two groups. In FG1 alliances were made between staff. In the second, alliances tended to be formed across the group between all members. In both groups alliances were either inter-practice between individuals from different practices or intra-practice – between individuals from the same practice.

### Between staff

Staff in FG1 would frequently support others as they described the perspective of their organisation. For example, the lead receptionist and practice manager from Practice 1 described the flexibility of their practice in meeting the needs of patients.

LR-1: I mean sometimes if somebody did come up, you know ‘I need a blood test and I really need one today’ then we will ask the nurse.

PM-1: Yeah, yeah.

LR-1: Then you know, it all depends on if the nurse has got time to do it or the health care assistant you know? On the whole at our practice, I mean if we did phone through and say ‘I need’ - you know – ‘I need you to do this’ they would do it

PM-1: Yeah the doctor or the nurses.

LR-1: Yeah, they are pretty good.

PM-1: Yeah, they’re pretty good our nurses. They’re quite flexible really

In the following exchange a patient questioned the attitude of staff members at Practice 1 in response the practice manager questioned the validity of the patient viewpoint supported by a staff member from another practice turning the conversation toward awkward patients, and suggesting that patient dissatisfaction was actually a function of the individual’s personality and independent of the service provided.

Patient one, practice one: I mean, they take a lot of flack, the receptionists. I mean, they do. For things that they can’t

PM-1: Yeah

Pt1-1: Alright you get the snotty ones, you know, I’m not saying who.

PM-1: I mean, we’re quite lucky with our patients, really, in that the majority are really good. But you do always remember the horrible ones. There’s no doubt about it, you know. You think ‘Oh God.’ But er, on the whole

Lead receptionist, practice two (LR-2): And some people just go through life being angry don’t they?

### Between staff and patients

The alliances formed in FG2 were across any patient and staff boundaries worked together, The more equal nature of the discussion described in the sociogram reflected a more evenly balanced conversation. In this example, the research nurse and a patient openly discussed how they would manipulate the system.

Res Nrs-3: but people will find a way round the system, so once they know the system, they’re smart enough to … work it out. Because they want to be seen they want the answer. I’ve done it I must admit when my daughter was younger and I wanted to book a session in case they weren’t able to give me an appointment later.

Patient, practice three (Pt-3): This is what frustrates me about the health service especially something like the NHS which is free at the point of care. Why do you need to know the system inside and out to use, to take advantage of it? Why is it not the same for everyone? It’s frustrating.

## Assumption of role within the group

In group discussions individuals can represent several viewpoints fulfilling one or a number of roles either as representatives of their external professional group, themselves as individuals, or as members of the four groups [11]. Across our focus groups there appeared a difference in the willingness to adopt alternative roles or perspectives. In FG1 roles appeared to be fixed from the beginning of the discussion. Within FG2 however staff participants were far more ready to acknowledge their experiences as patients.

### Fixed role assumption

Though some participants were invited as staff members, each was also a primary care patient and in both groups they were consistently invited to adopt the patient perspective by the moderator. However throughout FG1, staff members spoke only as a representative of the practice, in support of colleagues and current systems of practice. For example, when the idea of a software-based failsafe to alert staff if a result was delayed or lost, an idea suggested by patients previously, the practice manager spoke against the notion and felt it unnecessary.

PM-1: First of all most of them are going to come back anyway aren’t they? Second of all most patients will phone up or come in or ask, and the doctors are aware of the ones where there is an issue you know? Be it dementia, be it what…you know, learning difficulties whatever, so that’s the sort of third thing, and if it’s really serious the labs are phoning you up! Seems to me there are quite a lot of safety nets there.

### Fluid role assumption

In FG2, similar appeals were made to consider how existing systems might be amended to improve patient safety and experience. In response the office manager, though responsible for the administrative team at her practice, readily assumed the patient viewpoint. In this example, she agreed with a patient’s account of passivity in the presence of GPs and related her own experience of similar feelings despite her years of experience as an employee of a general practice surgery.

Pt-2: You go back to childhood in some way, especially when I get into hospital

OM-3: I mean I agree with what that gentleman said; to a certain extent it’s down to the patient; I work at a surgery so, you know, often I’m giving out results, but when I go to my own surgery as a patient I become this little girl who’s sort of. It’s true, you revert don’t you? I think, ‘well he must know what he’s doing so I’ll just wait for him to get back to me’.

A further example of a more fluid role assumption in FG2 was when the research nurse acknowledged concerns around the suitability of receptionists for communicating medical advice.

Res Nrs-3: The important thing is not the result but how you feel?

Several: Yeah.

Res Nrs-3: If you get a good result you think ‘well that’s it I can’t go back again because I’m normal’ and that is so, I feel, dangerous to the patient, because they should go back again; they should ask again.

## Discussion

### Summary of main findings

In our study that identified introduced changes to previously inconsistent systems for testing and communicating blood results in primary care we used mixed groups of patients and staff as part of the co-design process. We discovered that the specific constitution of each focus group affected how participants responded to each other and the moderator’s suggestions for change. Though outwardly the participants of the two groups appear broadly similar significant differences in the approach and output of each became apparent. These were manifest by the influence of social context, the various alliances that were formed between participants and the rigidity with which they adhered to their roles as practice staff or as patients. In the first group we unwittingly imported the organisational hierarchy that exists amongst administrative staff. The conversation that developed provided an interesting window on face-to-face interaction and discourse among these staff groups. However, staff participants assumed fixed roles of practice employees formed alliances with other staff members and defended current systems. This hampered attempts to determine which improvements suggested by staff and patients in a previous phase. The second focus group more closely met our intended aim of conducting an open discussion of emergent ideas. The dominant social context within what appeared a more equitable group was associational and any alliances were formed across staff and patient groups by staff seemingly more willing or able to adopt patient perspective.

### Specific findings

It’s widely acknowledged that informants in group settings do not treat their knowledge lightly, adjusting their contribution to fit the situation and the persons involved [13, 33, 34]. What people think and say depends on who is listening and how the question is posed, which alongside the pressures of social desirability [19–21] influences how individuals present themselves. These are an important consideration in collaborative co-designed service improvement methodologies that requires equity amongst wide-ranging groups of patients and staff of varying seniority. The weight of these influences between groups seemingly dependent upon the ratio of staff to patients but also the professional relationship between staff members.

In FG1, status appeared the central contextual influence on group interaction. The sociogrammatic analysis also illustrated the predominant role the Practice Manager took in the discussions. They responded to or were addressed by first to the majority of the moderator’s questions or comments reflecting previous evidence that those with a higher status in task-oriented groups assume leadership roles and tend to dominate the conversation [35, 36]. It is surprising that the best advice for collaborative service design does not acknowledge this [8] nor have any organisations or groups that have used co-designed service improvement [9, 10]. The practice manager appeared to assume responsibility for existing organisational boundaries and seemed reluctant to acknowledge any weaknesses in existing systems. The hesitancy of senior staff to accept alternative viewpoints has been observed previously [37]. In this instance, it may also be attributable to the complex organisational culture of larger primary care practices within which administrative staff which serve a key role in creating the boundary of the organisation [38–41]. This hierarchy is one way in which the organisational sub-culture seen in administrative groups [42] are typically dominated by rules, regulations, and might be involved in creating a top-down management hierarchy [43]. The staff alliances formed between the lead receptionist and practice manager in FG1 may be due to the important role a practice manager plays in the professional life of receptionists and the sense of worth receptionists gain from them [44]. That staff in FG2 though known to each other, did not work as part of the same team within the practice meant they were unencumbered by hierarchical considerations and together with patients were more willing to address suggestions raised by the moderator such as….

It has been proposed previously that conceptualisations of the self are not unitary but instead situational and can change depending upon the environment the individual finds themselves in [20, 45]. This appeared to be reflected in our group and has been observed previously in focus group participants [11]. In FG1 where status was the predominant contextual influence, all staff participants maintained the single role of representatives of the practice. In doing so they defended existing systems, despite requests from the moderator to consider alternative processes. However, within FG2, staff was far more ready to adopt user perspectives and consider change. This willingness to adopt or accept the perspectives of other stakeholders is the key rationale that underpins co-design [10].

### Future considerations

Patient and Public Involvement (PPI) is increasingly high on the agenda (Involve, 2012) [46] and is becoming mandatory for many funding bodies (National Institute for Health Research) [47]. Patients collaborating in mixed groups of this nature construct their “patient view” by establishing themselves as knowledgeable, or by validating or challenging another’s claims [48]. However, the degree to which they are comfortable in doing so can vary according to the dynamics of the particular group and our patient participants had very different experiences and offered contrasting levels of input apparently influenced by the constituency of the group. Within the status driven staff dominated FG1, organisational barriers were reinforced and patients had little opportunity to gain an independent voice. The hegemony was seldom challenged and the difficulties patients have in voicing criticisms of their care provider have been observed before [49]. This has serious implications for collaborative service improvement methodologies, particularly when used in discrete environments such as an individual primary care practice where contributors are frequently known to each other.

### Strengths and limitations

The impact of status and social context on group discussions has been widely acknowledged [17, 18] yet understanding their influence on the outcome of groups convened to redesign health care provision has been previously overlooked. Yet this understanding is critical if all staff participants are to be heard [15], and to prevent the patient voice being inadvertently stifled. The presence of senior clinical staff is likely to have altered the group dynamic but indicative of the growing pressure on general practice services [50] none were present and the profile of our groups is likely to be mirrored in future work in primary care. That staff participants in the second group were more willing to assume patient perspectives may in part be attributed to the intimacy of the smaller sized group, though this would not necessarily preclude status from becoming the dominant influence.

## Conclusions

To encourage balanced discussions in similar projects in primary care in the future the impact of status on both staff interaction and between staff and patients must be considered. Grouping combinations of patients and staff from the same practice needs to be carefully managed or avoided and where possible senior staff should be placed in groups separate to their immediate subordinates. Finally it’s worth noting that these effects might be ameliorated where all participants are fully engaged in the improvement project, understanding its rationale and methodology and so prepared to accept alternative perspectives and explore potential changes to existing systems.

## Abbreviations

EBCD: Evidence-Based Co-Design
FG1: Focus Group 1
FG2: Focus group 2
LR-1: Lead receptionist, practice one
LR-2: Lead receptionist, practice two
Mod: Moderator
OM-3: Office Manager-3
Pbt-3: Phlebotomist, practice three
PM-1: Practice manager
PPI: Patient and Public Involvement
Pt1–1: Patient one, practice one
Pt-2: Patient, practice two
Pt-3: Patient, practice three
Pt-4: Patient, practice 4
Res Nrs-3: Research nurse, practice three
